# Caffeine Supplementation for 4 Days Does Not Induce Tolerance to the Ergogenic Effects Promoted by Acute Intake on Physiological, Metabolic, and Performance Parameters of Cyclists: A Randomized, Double-Blind, Crossover, Placebo-Controlled Study

**DOI:** 10.3390/nu12072101

**Published:** 2020-07-16

**Authors:** Anderson Pontes Morales, Felipe Sampaio-Jorge, Thiago Barth, Anna Paola Trindade Rocha Pierucci, Beatriz Gonçalves Ribeiro

**Affiliations:** 1 Laboratory Research and Innovation in Sports Sciences, Federal University of Rio de Janeiro (UFRJ), Macaé, RJ 27930-560, Brazil; felipesjorge@gmail.com (F.S.-J.); ribeirogoncalvesb@gmail.com (B.G.R.); 2 Macaé Sports Secretary, City Government of Macaé (PMM), Macaé, RJ 27913-080, Brazil; 3 Higher Institutes of Education of CENSA (ISECENSA), Campos dos Goytacazes, RJ 28030-260, Brazil; 4 Postgraduate Program in Nutrition, Josué de Castro Nutrition Institute, Federal University of Rio de Janeiro (UFRJ), Macaé, RJ 21941-590, Brazil; appierucci@gmail.com; 5 Postgraduate Program in Bioactive Products and Biosciences, Federal University of Rio de Janeiro (UFRJ), Macaé, RJ 27930-560, Brazil; barththiago@yahoo.com.br; 6 Laboratory of Bioactive Products, Federal University of Rio de Janeiro (UFRJ), Macaé, RJ 27933-378, Brazil

**Keywords:** caffeine, endurance, exercise, fatigue

## Abstract

The present study investigated whether the caffeine supplementation for four days would induce tolerance to the ergogenic effects promoted by acute intake on physiological, metabolic, and performance parameters of cyclists. A double-blind placebo-controlled cross-over design was employed, involving four experimental trials; placebo (4-day)-placebo (acute)/PP, placebo (4-day)-caffeine (acute)/PC, caffeine (4-day)-caffeine (acute)/CC and caffeine (4-day)-placebo (acute)/CP. Fourteen male recreationally-trained cyclists ingested capsules containing either placebo or caffeine (6 mg∙kg^−1^) for 4 days. On day 5 (acute), capsules containing placebo or caffeine (6 mg∙kg^−1^) were ingested 60 min before completing a 16 km time-trial (TT). CC and PC showed improvements in time (3.54%, ES = 0.72; 2.53%, ES = 0.51) and in output power (2.85%, ES = 0.25; 2.53%, ES = 0.20) (*p* < 0.05) compared to CP and PP conditions, respectively. These effects were accompanied by increased heart rate (2.63%, ES = 0.47; 1.99%, ES = 0.34), minute volume (13.11%, ES = 0.61; 16.32%, ES = 0.75), expired O_2_ fraction (3.29%, ES = 0.96; 2.87, ES = 0.72), lactate blood concentration (immediately after, 29.51% ES = 0.78; 28.21% ES = 0.73 recovery (10 min), 36.01% ES = 0.84; 31.22% ES = 0.81), and reduction in expired CO_2_ fraction (7.64%, ES = 0.64; 7.75%, ES = 0.56). In conclusion, these results indicate that caffeine, when ingested by cyclists in a dose of 6 mg∙kg^−1^ for 4 days, does not induce tolerance to the ergogenic effects promoted by acute intake on physiological, metabolic, and performance parameters.

## 1. Introduction

The search for licit ergogenic resources is growing by cyclists in an attempt to minimize muscle fatigue generated in training sessions/competitions. In cyclists, muscle fatigue is manifested by the inability to repeatedly produce muscle strength or power over some time [1]. The ergogenic aid can be characterized as a supplement that delays fatigue, thus maintaining the output power and contributing to the improvement of sports performance [1,2]. In this scenario, caffeine (1, 3, 7-trimethylxanthine) has been described as a licit ergogenic resource that is effective in increasing performance in various sports (i.e., triathlon, and cycling) [1,3], in particular, sports that mainly involve muscle strength/power and aerobic endurance [1,2].

The primary mechanism of action described for caffeine is its role as a central nervous system (CNS) stimulant acting as a non-selective antagonist of the central and peripheral adenosine receptors (A_1_, A_2A_, A_2B_, and A_3_) [4]. Adenosine is a neurotransmitter that acts by modulating various physiological (i.e., perceptual and cardiorespiratory) and metabolic (i.e., cortisol and lactate blood levels) parameters.

In an attempt to establish the proper protocol to induce ergogenicity, researchers have examined different strategies, including the source of caffeine to be used [5], the dose used [2,3], and the acute time of ingestion [6]. Among these strategies, acute caffeine intake before exercise has been studied more closely by researchers [7,8,9,10,11].

The use of caffeine 1 h before exercise (acute intake) was suggested as an ergogenic strategy by the recommendation of current sports nutrition guidelines [12], using doses ranging from 3 to 6 mg∙kg^−1^ body mass. However, these recommendations do not address the impact of athletes’ usual caffeine consumption or chronic supplementation on their possible acute ergogenic effect [9,10]. For example, Beaumont et al. [9] observed that chronic use of caffeine supplementation (1.5 mg∙kg^−1^∙day^−1^) followed by acute intake (3 mg∙kg^−1^ body mass of caffeine) induced tolerance to ergogenic effects on performance. Lara et al. [10], using an incremental cycle ergometer model, demonstrated that daily caffeine supplementation (3 mg∙kg^−1^∙day^−1^) for 20 days, followed by acute intake (6 mg∙kg^−1^ body mass of caffeine) caused a reduction in the magnitude of ergogenicity on the ventilatory response. Both studies [9,10] used caffeine doses that exceeded the average amount of the subjects’ usual caffeine consumption and show that chronic use followed by an acute dose produced tolerance effects.

Irwin et al. [7] did not observe tolerance to ergogenic effects on time trial performance in low to moderate caffeine users. They received a dose (3 mg∙kg^−1^∙body weight) of caffeine similar to the usual consumption (3 mg∙kg^−1^∙day^−1^) in a short period (4 days), followed by an acute dose (6 mg∙kg^−1^ body weight of caffeine). This investigation [7] is comparable to the present study, but, differently, a higher dose of caffeine above the usual consumption will be tested in moderate to high consumers.

Thus, the present study investigated whether the caffeine supplementation for four days would induce tolerance to the ergogenic effects promoted by acute intake on physiological, metabolic, and performance parameters of cyclists.

## 2. Materials and Methods

### 2.1. Subjects

A statistical power analysis (G*Power 3.1) was performed to estimate the sample size based on the simulated cycling time trial (TT) (16 km) of a pilot study (*n* = 8 cyclists). The projected sample size was 12 with an effect size f of 0.41, alpha of 0.05, and power of 0.80. Using a more conservative approach (a priori) and taking into account possible dropouts, the effect size f was 0.36 (medium effect size), and the projected sample size was fourteen male cyclists. Participation was voluntary, and the following inclusion criteria were established: (a) all had at least 4 years of cycling experience; (b) participated in at least 20 competitions between 2018 and 2019; (c) have not had a history of cardiorespiratory, gastrointestinal, and musculoskeletal disorders in the last 3 months (Supplementary File—CONSORT Flow Diagram). They had a mean ± standard deviation (SD) age of 34.1 ± 4.4 years, a height of 178 ± 9 cm, body mass of 79.1 ± 11.8 kg, body mass index of 24.6 ± 2.1 kg∙m^2^, maximal oxygen uptake (VO_2_max) of 51.5 ± 6.3 mL∙kg^−1^∙min^−1^, and power output max (Wmax) of 398.9 ± 35.1 W. The training volume of the cyclists was 202 ± 83 km per week. In addition, a validated caffeine consumption questionnaire was administered to the participants, showing that all participants were moderate to high caffeine consumers (285.9 ± 108.0 mg∙day^−1^) [8]. Participants gave their written informed consent before inclusion. The protocol (2.540.958/2018) was approved by the Ethics Committee of Federal University of Rio de Janeiro (UFRJ) and was registered on a publicly accessible virtual platform (Brazilian Clinical Trials Registry) (http://www.ensaiosclinicos.gov.br/rg/RBR-5745nv/).

### 2.2. Study Design

**Figure 1:**
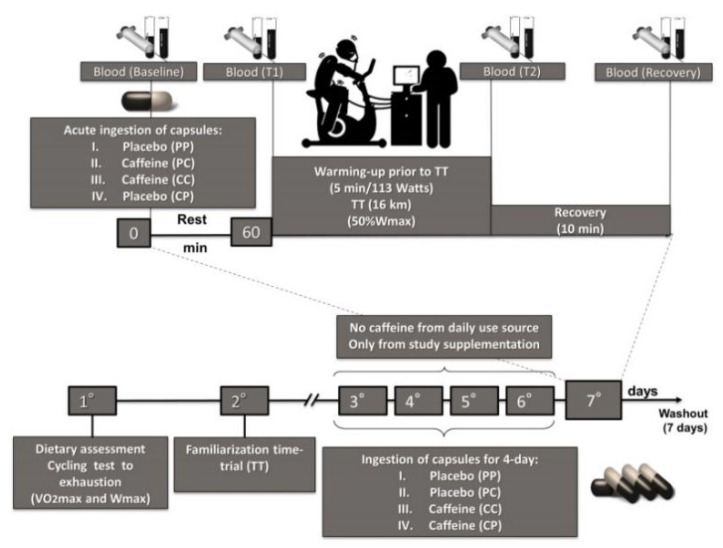
Experimental design.

A randomized, double-blind, crossover, placebo-controlled design was used in this study. On the first visit to the laboratory, participants underwent dietary assessment and cycling test to exhaustion. On the second visit, cyclists became familiar with the time trial test. So they received four visits from the researchers at home, one per day, for delivery and verification of capsule consumption (according to randomization). The other day they consumed the capsule acutely 60 min before the 16 km time trial test. The study preconized a seven-day washout [7,10] between the different intervention strategies characterized below. We tested the following strategies: Placebo–Placebo (PP), participants received Placebo (4-day supplementation), and Placebo (acute ingestion, 60 min before simulated cycling TT completed). Placebo capsules were 250 mg of magnesium silicate single daily dose. Placebo–Caffeine (PC), participants received Placebo (4-day supplementation), and Caffeine (acute ingestion, 60 min before simulated cycling TT completed). Caffeine capsules were 6 mg∙kg^−1^ body mass. Caffeine–Caffeine (CC), participants received Caffeine (4-day supplementation), and Caffeine (acute ingestion, 60 min before simulated cycling TT completed). Caffeine–Placebo (CP), participants received Caffeine (4-day supplementation), and Placebo (acute ingestion, 60 min before simulated cycling TT completed). Random numbers placed in sealed envelopes drawn only by the second researcher constituted the randomization process of the study. At the first visit to the laboratory, the researchers verified the routine energy and caffeine intake of food, VO_2_max, and workload capacity in the graded test until exhaustion in the cycle ergometer. The athletes were instructed to withdraw all their caffeine consumption (i.e., food sources of caffeine) during the experiment and were monitored by telephone contact, e-mail, and in-person. On the test day, cyclists arrived fasting in the laboratory, and soon an intravenous cannula (20G Jelco; B. Braun Medical Inc., Bethlehem, PA, USA) was inserted into the forearm, and then four blood samples (10 mL) were obtained: before ingestion of capsules (baseline), 60 min after intake of capsules (T1), immediately after TT (T2) and after 10 min of recovery (recovery). Cyclists did not exercise 24 h before the experimental trials in the laboratory. The athletes were instructed to continue the routine of daily training. The experimental trials were performed at the same time of day (7:00 AM) (Figure 1).

### 2.3. Habitual Food Intake Recording and Caffeine-Containing Foods

Each athlete completed the 24-h food record from the first to the fourth trial. According to the 24-h food record of the first trial, it was photocopied and returned to athletes so that the same diet could be repeated for subsequent trials. Energy consumption, total carbohydrates, total proteins, and total lipids were determined. The TACO^®^ database was used to quantify macronutrient intake and Dietpro^®^ 5i (Dietpro, Viçosa, MG, Brazil) software was used for nutrient calculation. To assess habitual caffeine intake, we used a questionnaire adapted from Landrum et al. [13]. The questionnaire was applied individually with the supervision of a qualified nutritionist. The questionnaire consists of a list of food sources of caffeine (coffee, tea, cocoa, chocolate, soft drinks, medicine, and dietary supplements) and the period of the day (morning, 6:00 AM to 12 noon; afternoon, 12 noon to 6:00 PM; evening, 6:00 AM to 2:00 AM; night, 2:00 AM to 6:00 AM) consumed. The types of food, dietary supplements in the diet, and medicaments of athletes who contained caffeine were identified. Caffeine content was obtained from the USDA Food Composition Databases, from food labels and medication package inserts.

### 2.4. VO_2_max and Workload Capacity

Participants performed a graded exercise test until exhaustion on a mechanically braked cycle ergometer (Biotec 2100, Cefise^®^, São Paulo, Brazil) to determine VO_2_max. Participants began pedaling at a power output of 113 W, with 45-W increments every 2 min and pedaling rate 88 rpm was kept constant until exhaustion. Maximal workload capacity (Wmax) was determined by the following equation: Wmax = Output Power (Woutput) + ((t/113) × 45), Woutput is the workload in the last completed stage, and t is the time spent in the final stage not completed [8]. Heart rate was monitored continuously (Polar Electro Oy^®^, Kempele, Finland). Pulmonary gas exchange was determined breath by breath, by use of a gas analysis system VO2000 (MedGraphics^®^, St. Paul, MN, USA). The equipment was calibrated automatically according to the manufacturer’s specifications before each test. The present study determined and validated the VO_2_max following these criteria: increase in VO_2_ less than 2.1 mL∙kg^−1^∙min^−1^ by increasing the intensity; exhaustion of the individual; the respiratory exchange ratio bigger than 1.10. The plateau in VO_2_ was determined when the difference in oxygen consumption in the final 30 s of the last two stages (∆VO_2_) was ≤ 2.1 mL∙kg^−1^∙min^−1^.

### 2.5. Simulated Cycling TT Performance

Participants reported to the laboratory at individually standardized times after an 8-h fasting period. Before the main exercise, participants underwent a 5-min warm-up at 113 W (88 rpm), immediately followed by the simulated cycling TT. The protocol consisted of a continuous test, with each subject cycling with a rotation higher than 88 rpm as quickly as possible at a distance of 16 km at work intensity. It was used a constant workload representing 50% of the maximum capacity (above 199.67 ± 17.90 W equivalent to work higher than ~407 kJ, determined by the following equation: work (J) = 50% Wmax × t) on the mechanized cycle ergometer brake. The cycle ergometer was connected to a laptop using “Ergometric” software (version 7.0, Cefise^®^, São Paulo, Brazil) for the collection and storage of data, such as power output (W) and cadence (rpm). The participants were blinded to performance-related information (exercise time and cadence) during the tests. The only information that the participants received during the test was the distances (2 km, 4 km, 6 km, 8 km, 10 km, 12 km, 14 km, and 16 km). At these set intervals during the trial, participants were asked their rating of perceived exertion (RPE) using the 0-to-10-point Borg Scale [14]. Ventilation (volume minute V_E_, oxygen uptake VO_2_, carbon dioxide output VCO_2_, O_2_ expiration fraction FeO_2_, and CO_2_ expiration fraction FeCO_2_), heart rate (HR), and power output parameters were measured continuously during the TT. The parameters listed are expressed in ten points (10%, 20%, 30%, 40%, 50%, 60% 70%, 80%, 90%, and 100%), the curve of the total time completed during the TT and average in PP, PC, CC and CP conditions. All the trials were performed in a climate-controlled laboratory (21 °C to 24 °C, 41% to 52% relative humidity).

### 2.6. Nutritional Intervention

The participants were administered a dose of anhydrous caffeine (6 mg∙kg^−1^ body mass) or placebo (250 mg magnesium silicate), provided in gelatin capsules, identical in color, size, and appearance. Because the participants are used to consuming average amounts of caffeine daily, around 285.92 ± 108.04 mg∙day^−1^ (verified through a questionnaire), the present study chose to offer a higher dose than usual. This dose (6 mg∙kg^−1^ of body weight) represented a dosage of 474.78 ± 70.80 mg, exceeding the average amount of usual consumption, which made it possible to verify the effects of tolerance. In the presence of a researcher, all athletes were instructed to take a single capsule daily at the same time (9:00 AM) during the 4-day supplementation. In acute ingestion, the capsule was administered with 250 mL of water 60 min before TT. Participants rested quietly for 60 min before starting the test in all the sessions. Supplements for each participant were prepared and separated by a non-affiliated researcher to ensure double-blinding.

### 2.7. Blood Caffeine, Cortisol, Lactate, and Glucose Concentrations Analysis

The measurement of blood levels of caffeine, cortisol, lactate, and glucose were performed at baseline, T1, T2, and recovery. Plasma and serum were obtained by centrifugation at 2.500 rpm at 4 °C for 20 min. The resultant plasma and serum were stored at −20 °C until the analyses could be performed. The caffeine blood levels were determined using a HPLC method, adapted from Ribeiro et al. [2]. The HPLC analyses were carried out using a Shimadzu chromatograph (Shimadzu^®^ Corp., Kyoto, Japan). To perform the lactate and glucose analyses, commercial kits from (Labtest, Lagoa Santa, Brazil) and the BIO200 analyzer (Bioplus^®^, São Paulo, Brazil), respectively, were used. Cortisol was analyzed by the chemiluminescent technique using the reagent kit (Abbott Diagnostics Division, Santa Clara, CA, USA) and the Architect i2000SR automatic immunoassay analyzer (Abbott^®^, Abbott Park, IL, USA).

### 2.8. Statistical Analysis

Data are expressed as mean ± SD or as median (interquartile deviation), following the normality of Shapiro Wilk test. All variables were analyzed (SPSS, version 16.0) with one way or two way ANOVA according to the data. Mauchly’s test of sphericity was performed for all test variables, and Greenhouse–Geisser correction for within-subject effects was used in cases where the assumption of sphericity was violated. Significant interactions were followed up by pairwise comparisons through simple main effect analysis with Tukey correction for multiple comparisons (habitual food intake recording, perception of effort responses and cardiorespiratory parameters, cycling TT and power output, cortisol and lactate concentrations). We used the Friedman and Kruskal–Wallis tests for the glucose and caffeine concentrations. Effect Sizes (ES) (Cohen’s d: small effect ≥ 0.20, medium effect ≥ 0.50, large effect ≥ 0.80, very large effect ≥ 1.30) and Delta (Δ) of caffeine supplementation were calculated as the difference between means corresponding to caffeine (PC, CC, CP) and placebo (PP). For all statistical analyses, a *p* value less than 0.05 was considered statistically significant.

## 3. Results

### 3.1. Habitual Food Intake Recording

Food intake, which did not exhibit significant main effects in treatment 24 h before the trials, is shown in Table 1: energy [F(2.320, 30.16) = 0.8352, *p* = 0.4590], carbohydrates [F(1.901, 24.71) = 1.117, *p* = 0.3406], protein [F(1.933, 25.13) = 2.097, *p* = 0.1450], and lipids [F(1.809, 23.52) = 1.912, *p* = 0.1729].

### 3.2. RPE and Cardiorespiratory Parameters

RPE only exhibited significance of distance traveled in TT (Table 2) [F(7, 91) = 150.1, *p* = 0.0001] for the main effect. There were no differences between treatments and interaction between treatment and distance traveled in TT (*p* > 0.05) (Table 2).

**Figure 2:**
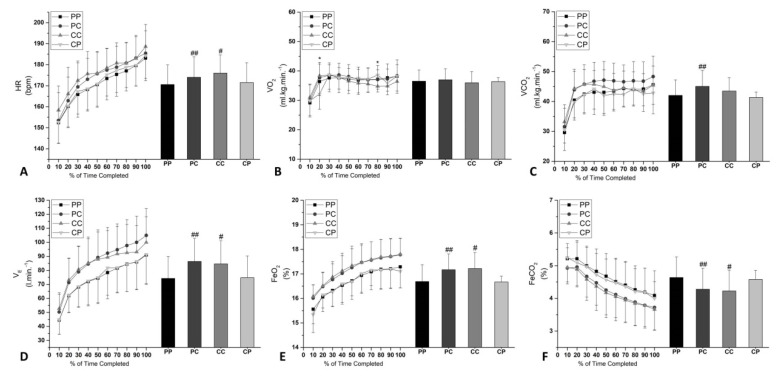
Mean (±SD). Mean (±SD). Curve values and the average of cardiorespiratory parameters during TT performance: (**A**) HR, (**B**) VO_2_, (**C**) VCO_2_, (**D**) V_E_, (**E**) FeO_2_, and (**F**) FeCO_2_. * Significant difference to CC from CP (*p* <0.05). ^#^ Significant difference from CP (*p* < 0.05). ^##^ Significant difference from PP (*p* < 0.05).

HR (Figure 2A) showed significant treatment [F(3, 39) = 9.134, *p* = 0.000] and percentage of time curve [F(9, 117) = 109.0, *p* = 0.000] in the main effect. However, there was no significance observed in the interaction between treatment and percentage of time curve [F(27, 351) = 1.345, *p* = 0.120]. HR was higher with caffeine in the PC condition (174.01 ± 9.94 vs. 170.6 ± 9.51 bpm) (Δ = 3.41 bpm; *p* = 0.02; ES = 0.34 [95% CI 0.36, 6.46]) than that with PP, and higher in the CC condition (176.01 ± 9.59 vs. 171.49 ± 9.47 bpm^−1^) (Δ = 4.52 bpm; *p* = 0.00; ES = 0.47 [95% CI 1.32, 7.42]) than that with CP. No differences were observed between CC and PC (*p* > 0.05).

There was no significant difference (*p* = 0.7947) in the main effects on VO_2_ (Figure 2B). However, a significant percentage of time curve [F(9, 117) = 14.37, *p* = 0.0001] and interaction between treatment and percentage of time curve (*p* = 0.0005) effects were seen.

VCO_2_ (Figure 2C) exhibited significant treatment [F(3, 39) = 5.065, *p* = 0.0047] and percentage of time curve [F(9, 117) = 38.33, *p* = 0.001] main effects. However, there was no significance observed in the interaction between treatment and percentage of time curve (*p* = 0.7184). VCO_2_ was higher with caffeine in the PC condition (45.06 ± 5.32 vs. 42.04 ± 5.15 mL∙kg^−1^∙min^−1^) (Δ = 3.02 mL∙kg^−1^∙min^−1^; *p* = 0.02; ES = 0.57 [95% CI 0.24, 5.79]) than that with PP. No differences were observed between CC and PC (*p* > 0.05).

V_E_ (Figure 2D) exhibited significant treatment [F(3, 39) = 14.49, *p* = 0.000] and percentage of time curve [F(9, 117) = 76.07, *p* = 0.000] main effects. However, a significant interaction between treatment and percentage of time curve (*p* = 0.070) was not observed. VE was higher with caffeine in the PC condition (86.38 ± 16.50 vs. 74.26 ± 15.62 L∙min^−1^) (Δ = 12.12 L∙min^−1^; *p* = 0.00; ES = 0.75 [95% CI 5.76, 18.47]) than that with PP, and with caffeine in the CC position (84.62 ± 16.44 L∙min^−1^ vs. 74.81 ± 15.44 L∙min^−1^) (Δ = 9.81 L∙min^−1^; *p* = 0.00; ES = 0.61 [95% CI 3.44, 16.16]) than that with CP. No differences were observed between CC and PC (*p* > 0.05).

FeO_2_ (Figure 2E) exhibited significant treatment [F(3, 39) = 10.93, *p* = 0.0001] and percentage of time curve [F(9, 117) = 91.09, *p* = 0.0001] main effects. However, there was no significant interaction between treatment and percentage of time curve (*p* = 0.9996) observed. FeO2 was higher with caffeine in the PC condition (17.17 ± 0.65% vs. 16.69 ± 0.68%) (Δ = 0.48%; *p* = 0.00; ES = 0.72 [95% CI 0.07, 0.88]) than that with PP, and in the CC condition (17.22 ± 0.65% vs. 16.67 ± 0.24%) (Δ = 0.55%; *p* = 0.00; ES = 0.96 [95% CI 0.20, 0.88]) than that with CP. No differences were observed between CC and PC (*p* > 0.05).

FeCO_2_ (Figure 2F) exhibited significant treatment [F(3, 39) = 8.006, *p* = 0.0003] and percentage of time curve [F(9, 117) = 60.46, *p* = 0.0001] main effects. However, there was no significant interaction between treatment and percentage of time curve (*p* = 0.9996) observed. FeCO2 was lower with caffeine in the PC condition (4.28 ± 0.64% vs. 4.64 ± 0.63%) (Δ = −0.36%; *p* = 0.00; ES = 0.56 [95% CI −0.64, −0.07]) than that with PP, and with caffeine in the CC condition (4.23 ± 0.63% vs. 4.58 ± 0.28%) (Δ = −0.35%; *p* = 0.00; ES = 0.64 [95% CI −0.63, −0.07]) than with CP. No differences were observed between CC and PC (*p* > 0.05).

### 3.3. Power Output and Cycling TT Performance

**Figure 3:**
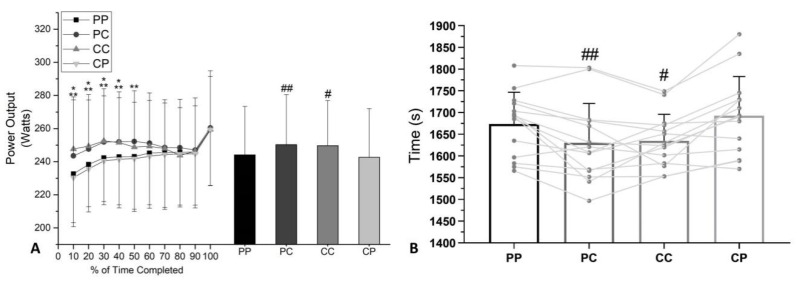
Mean (±SD). Curve values and the average of performance parameters during TT performance: (**A**) Power output and (**B**) time of simulated cycling TT performance. * Significant difference to CC from CP (*p* < 0.05). ** Significant difference to PC from PP (*p* < 0.05). ^#^ Significant difference from CP (*p* < 0.05). ^##^ Significant difference from PP (*p* < 0.05).

Power output (Figure 3A) exhibited significant treatment [F(3, 39) = 8.957, *p* = 0.0001] and percentage of time curve [F(9, 117) = 9.409, *p* = 0.0001], and an interaction between treatment and percentage of time curve [F(27, 351) = 2.383, *p* = 0.0002] main effects. Power output was higher with caffeine in the PC condition (250.3 ± 30.24 W vs. 244.1 ± 29.33 W) (Δ = 6.20 W; *p* = 0.00; ES = 0.20 [95% CI 1.25, 11.10]) than that with PP, and in the CC condition (249.7 ± 27.23 W vs. 242.5 ± 29.51 W) (Δ = 7.12 W; *p* = 0.00; ES = 0.25 [95% CI 0.55, 10.53]) than that with CP. No differences were observed between CC and PC (*p* > 0.05).

There was a main effect of treatment on TT performance (Figure 3B) [F(2.488, 3234) = 11.66, *p* = 0.0001]. TT test completion time was lower with caffeine in both PC (1631 ± 90.45 s vs. 1674 ± 72.88 s) (Δ = −43 s; *p* = 0.04; ES = 0.51 [95% CI −85.00, −1.71]) than that with PP, and in CC (1634 ± 61.29 s vs. 1692 ± 90.47 s) (Δ = −58 s; *p* = 0.00; ES = 0.72 [95% CI −97.38, −18.34]) than that with CP. No differences were observed between CC and PC (*p* > 0.05).

### 3.4. Blood Caffeine, Cortisol, Lactate and Glucose Concentrations

Caffeine concentration exhibited significant time (*p* = 0.0001) and treatment (*p* = 0.0001) main effects. Cortisol concentration only exhibited significant time [F(3, 39) = 163.4, *p* = 0.000] main effect. Lactate concentration exhibited significant treatment [F(3, 39) = 18.32, *p* = 0.000] and time [F(3, 39) = 239.6, *p* = 0.000], and an interaction between treatment and time [F(9, 117) = 11.61, *p* = 0.000] main effects. Glucose concentration exhibited significant time (*p* = 0.0001) main effect (Table 3).

## 4. Discussion

The results of the present study indicate that caffeine, when ingested by cyclists in a dose of 6 mg∙kg^−1^ for 4 days, does not induce tolerance to the ergogenic effects promoted by acute intake on physiological, metabolic, and performance parameters. Furthermore, CC and PC showed improvements in time and in output power compared to CP and PP conditions, respectively. The physiological and metabolic responses such as an increase in heart rate, minute volume, expired O_2_ fraction, blood lactate concentration (T2 and Recovery), and reduction in expired CO_2_ fraction were similar in both withdrawal and 4-day supplementation conditions.

The cyclists in the present study who consumed caffeine acutely (CC and PC) had more output power in the first half of the TT test (Figure 3A). It can be explained by the effect of caffeine to pre-dispose the subject to perform tasks [4,12,15]. With this, the individuals achieved a significant improvement in the time of the TT test, indicating a potential ergogenic effect corroborating with previous studies [1,8]. The present findings are in agreement with the results of Irwin et al. [7]. They reported that acute caffeine intake (6 mg∙kg^−1^ body mass of caffeine) significantly improves exercise performance, regardless of whether a period of withdrawal or consumption (3 mg∙kg^−1^ body mass of caffeine) for 4 days is imposed on regular users of caffeine.

According to the authors [7], an explanation for the inability to influence the ergogenic potential of caffeine in their study would be the participants’ usual consumption (3 mg∙kg^−1^∙day^−1^). In another study [8], the authors observed similar improvements in performance using a cross-sectional protocol, comparing low (58 ± 29 mg∙day^−1^ or 0.80 mg∙kg^−1^∙day^−1^), moderate (143 ± 25 mg∙day^−1^ or 1.90 mg∙kg^−1^∙day^−1^) and high (351 ± 139 mg∙day^−1^ or 4.91 mg∙kg^−1^∙day^−1^) consumers, when administered in cyclists an acute dose of 6 mg∙kg^−1^ body mass of caffeine. The plausible explanation for this finding indicates that the dose used for acute intake exceeded the average amount of caffeine about the usual consumption of low, moderate, and high consumers [8]. In our study, cyclists were moderate to high consumers, and the dose of 6 mg∙kg^−1^ body mass of caffeine exceeded the average amount of usual caffeine consumption. Thus, in the present study, it was suggested that the improvement in performance is related to acute intake, regardless of habitual consumption and caffeine supplementation for 4 days.

A previous study by Graham and Spriet [16] concluded that an acute intake of 6 mg∙kg^−1^ body mass of caffeine could cause saturation in hepatic metabolism and, consequently, the maintenance of caffeine blood concentration for a prolonged time. Therefore, we believe that the improvement in time (Figure 3B) observed in 85.71% (CC and PC) of cyclists (12 cyclists) may be associated with the maintenance of the blood caffeine concentration over time (~1 h 37 min) (Table 3). These results reinforce the notion that the use of this dose (6 mg∙kg^−1^ body mass) may reduce interindividual metabolic responses [8].

Furthermore, evidence shows that an increase in primary caffeine metabolites (paraxanthine, theobromine, and theophylline) is due to caffeine metabolism, which may promote tolerance effects [15,17,18]. Because they have a higher affinity for adenosine receptors than caffeine [19], this could result in the higher development of tolerance to caffeine’s ergogenic effects [9,10]. To reverse the effects of tolerance, the use of the caffeine withdrawal strategy, followed by the use of an acute dose, may result in the resensitization of adenosine receptors (A_2A_/A_2B_) [7,15]. However, in the present study, cyclists’ blood caffeine concentrations (verified in acute intake) remained similar between trials (CC and PC) (Table 3), suggesting that the ergogenic effects observed in the CC and PC conditions were due to acute intake (use before the test). Previous evidence [7,15] suggested that withdrawal caffeine intake strategy a few days before the competition is needed to elicit an ergogenic effect of caffeine. However, the present data propose otherwise. It seems that athletes may (at least over the short-term) continue using caffeine before the competition.

The effects of acute caffeine intake appear to regulate blood cortisol and glucose concentrations during exercise [20,21]. From this perspective, elevated blood cortisol concentrations may stimulate gluconeogenesis in the liver and induce substantial increases in blood glucose levels. There is evidence that caffeine acts by increasing cortisol secretion by increasing adrenocorticotropic hormone (ACTH) production in the pituitary [21], although the precise mechanisms still need to be characterized. The findings of the present study indicated that caffeine intake did not alter blood cortisol and glucose concentrations between experimental trials (Table 3). However, the increase in cortisol and glucose concentration observed at T2 and recovery (Table 3), regardless of caffeine intake, may be associated with exercise using intensities ≥40% of VO_2_max [22], as shown in our results where cyclists reached an average intensity of approximately 70.61% of VO_2_max.

The distribution of adenosine receptors throughout the peripheral tissues and the central nervous system suggests that caffeine may act directly or indirectly through multiple mechanisms. The findings of previous studies [23,24] showed higher HR responses during exercise, which is due to the ergogenic effects promoted by acute caffeine intake. These findings suggest that caffeine ingestion attenuates the brainstem response of the baroreflex due to metaborreflex activation caused by the accumulation of muscle metabolites (lactate, H^+^, and P_i_) as a result of increased muscle metabolism during exercise. Thus, we attributed the increase in HR to the high blood lactate concentrations observed after TT (T2 and recovery) (Table 3). It was then expected, due to changes in CNS responses and peripheral metabolism (metabolic muscle accumulation), that acute caffeine intake may reduce RPE [3]. In the present study, however, probably due to the exercise protocol used (TT) [25], this assumption was not observed (Table 2). According to the findings of the study [26] that investigated the effects of caffeine intake on neuromuscular fatigue, the reduction in RPE is strongly associated with the use of exercise protocols until exhaustion.

Adopting a high pacing strategy at the beginning of TT is the most commonly used form by cyclists expecting to increase fatigue tolerance [27]. However, success in using this strategy is reduced when many highly trained cyclists exhibit induced arterial hypoxemia (at sea level) at the beginning of exercise [28]. This phenomenon is explained by the delayed physiological response of integrated neural control, because the reduction in the ventilatory mechanism may negatively impact the release of O_2_ to the muscles. Interestingly, Jones et al. [27] observed in cyclists that the reduction in the percentage of the mean response time of the ventilatory mechanism was significantly correlated (r = 0.85, *p* < 0.05) with the improvement in time to TT. Investigators [27,28] argue that the primary focus on reducing the effects of hypoxemia is associated with increased respiratory medullary complex stimulation. This evidence suggests that acute caffeine intake may increase the sensitivity of peripheral chemoreceptors to potentiate hyperventilation (minute ventilation) during exercise [28,29]. Thus, our findings indicate that the improvement in the partial pressure of alveolar oxygen (Figure 2D–F) induced by acute caffeine intake may be associated with an increase in the output power curve at the beginning of TT (Figure 3A).

In future studies, it should be encouraged to test whether the blinding procedure was effective. Asking participants to identify the supplement ingested, to test the efficacy of the blinding method, should be a great way to avoid bias in the analysis of the results [30]. The experimental design used in the present investigation presented some limitations that should be discussed for the correct application of the results. In the present study, the cycle ergometer was used to determine the ergogenic effects of caffeine under laboratory conditions. However, these results should be confirmed in additional research protocols, which use field tests (i.e., cycling competition or simulations) or assessments utilizing the athlete’s bike, to transfer the findings of this investigation to coaches accurately. Other limitations of the present study are related to the interindividual variability observed in caffeine metabolism [17] and to the analysis of the expression of adenosine receptors in skeletal muscle tissue, given its up-regulation during chronic treatment with the use of caffeine [31]. According to Ribeiro et al. [1], genetic polymorphisms in related genes to caffeine metabolism, such as aryl-hydrocarbon receptor (AHR) and cytochrome P450 1A1 and 1A2 (CYP1A1-CYP1A2, Prenyl (Decaprenyl)), are a potential explanation for the variability in the ergogenic response to caffeine supplementation in trained athletes. Given these prior findings, it could be hypothesized that a higher metabolism would be advantageous for maximizing the ergogenic benefit of caffeine [18,32]. However, we were not able to evaluate the polymorphisms in genes related to caffeine metabolism, which could minimize the impact on the interindividual variability of cyclists.

## 5. Conclusions

In conclusion, these results indicate that caffeine, when ingested by cyclists (moderate to high caffeine consumers) in a dose of 6 mg∙kg^−1^ for 4 days, does not induce tolerance to the ergogenic effects promoted by acute intake on physiological, metabolic, and performance parameters. Furthermore, the ergogenic effects observed in the supplementation and withdrawal conditions for 4 days were due to acute intake, verified by a similar blood concentration of caffeine.

## Figures and Tables

**Table 1:** Food intake of cyclists 24-h before the trials.

	PP (*n* = 14)	PC (*n* = 14)	CC (*n* = 14)	CP (*n* = 14)
Energy (kcal)	2132.93 ± 743.67	2056.90 ± 389.76	2181.42 ± 452.35	2261.41 ± 525.69
Carbohydrates (g∙day^−1^)	250.92 ± 67.58	242.06 ± 67.23	276.70 ± 80.51	291.61 ± 67.58
Protein (g∙day^−1^)	121.62 ± 45.38	114.38 ± 42.55	126.62 ± 47.25	131.63 ± 45.38
Lipids (g∙day^−1^)	78.50 ± 45.69	69.23 ± 25.28	62.01 ± 27.92	76.35 ± 27.62

PP, Placebo–Placebo; PC, Placebo–Caffeine; CC, Caffeine–Caffeine; CP, Caffeine–Placebo.

**Table 2:** Mean (±SD) rating of perceived exertion (RPE) for every 2 km of distance traveled in cycling time trial (TT).

	Distance							
Treatment	2 km	4 km	6 km	8 km	10 km	12 km	14 km	16 km
PP	3.56 ± 1.01	4.67 ± 1.22 ^a^	5.67 ± 1.32 ^a^	6.44 ± 1.13 ^a^	6.89 ± 1.05 ^a^	8.11 ± 0.93 ^a^	9.00 ± 1.00 ^a^	9.11 ± 0.78 ^a^
PC	3.44 ± 1.51	4.67 ± 1.32 ^a^	5.78 ± 1.48 ^a^	6.56 ± 1.42 ^a^	7.11 ± 1.05 ^a^	8.00 ± 1.12 ^a^	8.78 ± 0.97 ^a^	9.00 ± 1.0 ^a^
CC	4.00 ± 1.32	4.67 ± 1.12 ^a^	5.56 ± 1.42 ^a^	6.67 ± 1.22 ^a^	7.22 ± 0.83 ^a^	8.00 ± 1.00 ^a^	8.67 ± 1.32 ^a^	8.78 ± 1.30 ^a^
CP	4.11 ± 1.54	5.22 ± 1.72 ^a^	6.22 ± 1.64 ^a^	7.00 ± 1.41 ^a^	7.44 ± 1.13 ^a^	8.67 ± 1.12 ^a^	9.00 ± 1.00 ^a^	9.11 ± 0.93 ^a^

PP, Placebo–Placebo; PC, Placebo–Caffeine; CC, Caffeine–Caffeine; CP, Caffeine–Placebo. ^a^ Significant difference from 2 km (*p* < 0.05).

**Table 3:** Analysis of blood samples (*n* = 14). Mean ±SD and median (interquartile deviation).

	Baseline	T1	T2	Recovery
Caffeine (µg·mL^−1^)				
PP	0.34 (0.14)	0.37 (0.22)	0.36 (0.16)	0.37 (0.11)
PC	0.00 (0.32)	7.60 (1.32) ^a,b^	7.61 (1.33) ^a,b^	7.80 (0.56) ^a,b^
CC	0.45 (0.41)	8.19 (0.76) ^a,c^	8.35 (0.76) ^a,c^	8.18 (1.11) ^a,c^
CP	0.33 (0.36)	0.30 (0.38)	0.21 (0.31)	0.27 (0.44)
Cortisol (µg·mL^−1^)				
PP	10.74 ± 3.45	10.98 ± 3.34	14.90 ± 4.2 ^a^	15.33 ± 4.20 ^a^
PC	9.64 ± 3.66	9.70 ± 3.67	16.60 ± 5.2 ^a^	16.84 ± 5.06 ^a^
CC	10.73 ± 3.58	10.90 ± 3.39	16.02 ± 4.28 ^a^	16.44 ± 4.23 ^a^
CP	11.49 ± 2.14	11.62 ± 2.26	15.76 ± 2.61 ^a^	16.17 ± 2.80 ^a^
Lactate (mmol·L^−1^)				
PP	1.09 ± 0.42	1.02 ± 0.50	12.58 ± 4.59 ^a^	9.16 ± 3.31 ^a^
PC	1.04 ± 0.60	1.38 ± 0.54	16.13 ± 5.06 ^a,b^	12.02 ± 3.68 ^a,b^
CC	1.11 ± 0.48	1.30 ± 0.53	15.14 ± 4.34 ^a,c^	12.01 ± 4.07 ^a,c^
CP	1.14 ± 0.77	1.01 ± 0.64	11.69 ± 4.43 ^a^	8.83 ± 3.35 ^a^
Glucose (mmol·L^−1^)				
PP	5.84 (1.62)	5.67 (1.35)	9.80 (4.25) ^a^	9.87 (3.31) ^a^
PC	5.41 (1.79)	5.72 (1.33)	12.10 (6.05) ^a^	12.55 (6.07) ^a^
CC	5.97 (1.37)	5.97 (1.46)	10.82 (2.45) ^a^	10.80 (4.33) ^a^
CP	6.44 (1.76)	5.94 (1.33)	9.54 (4.34) ^a^	9.66 (3.31) ^a^

PP, Placebo–Placebo; PC, Placebo–Caffeine; CC, Caffeine–Caffeine; CP, Caffeine–Placebo. T1—60 min after capsule ingestion. T2—immediately after TT performance. ^a^ Significant difference from baseline or/and T1 (*p* < 0.05). ^b^ Significant difference from PP (*p* < 0.05). ^c^ Significant difference from CP (*p* < 0.05).

## Supplementary Materials

The following are available online at https://www.mdpi.com/2072-6643/12/7/2101/s1, Figure S1: Disposition of study participants.
