# Supplementary figures and images for: Caffeine Supplementation for 4 Days Does Not Induce Tolerance to the Ergogenic Effects Promoted by Acute Intake on Physiological, Metabolic, and Performance Parameters of Cyclists: A Randomized, Double-Blind, Crossover, Placebo-Controlled Study
Source: Nutrients. 2020 Jul 16;12(7):2101. doi: 10.3390/nu12072101 (PMC7400874; doi:10.3390/nu12072101)

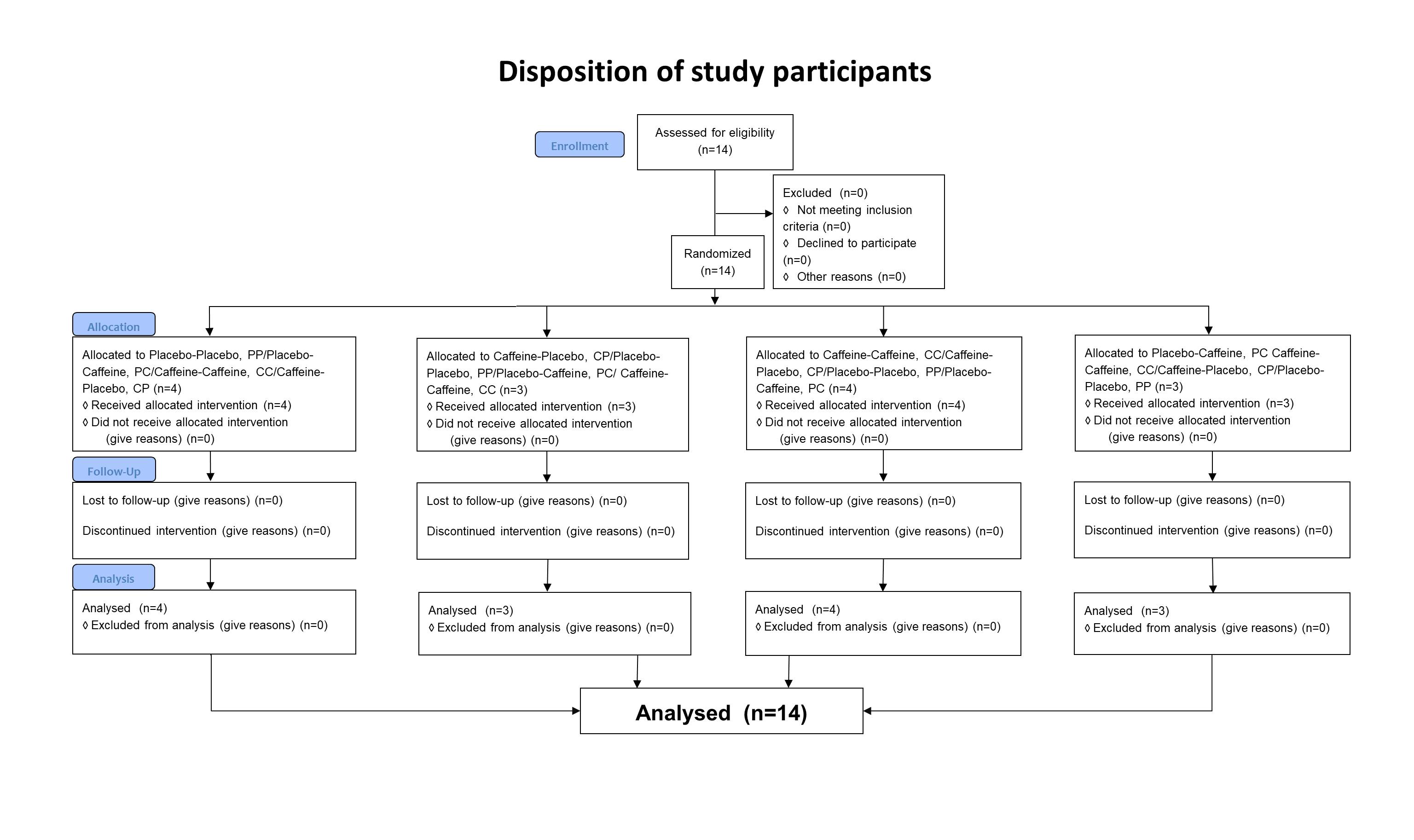

Supplement: Supplementary file 1 [file nutrients-12-02101-s001.zip › nutrients-852739-supplementary.jpg]
